# Artery of Percheron Infarction Presenting As Altered Consciousness in an Elderly Patient: A Case Report

**DOI:** 10.7759/cureus.87588

**Published:** 2025-07-09

**Authors:** Saad Ansar, Reham Umer, Chathura Madhushan Angulugaha Angulugaha Gamage, Muhammad Sharjeel, Zara Ammer

**Affiliations:** 1 General Medicine, Peterborough City Hospital, North West Anglia NHS Foundation Trust, Peterborough, GBR; 2 Geriatric Medicine, Peterborough City Hospital, North West Anglia NHS Foundation Trust, Peterborough, GBR; 3 Acute Medicine, East Surrey Hospital, Redhill, GBR; 4 Internal Medicine, Mid City Hospital Multan, Multan, PAK

**Keywords:** artery of percheron, bilateral thalamic infarction, mri diagnosis, neuroimaging, stroke

## Abstract

Bilateral thalamic infarcts represent an uncommon manifestation of acute ischemic stroke, often resulting from occlusion of the artery of Percheron (AOP). Thereby, we report the case of an 86‐year‐old female with a background of multiple comorbidities who presented with sudden-onset unresponsiveness. Her Glasgow coma scale (GCS) score on admission was 10/15. Non-contrast computed tomography (NCCT) of the head ruled out intracranial hemorrhage. While being empirically managed for a suspected meningitis, the patient’s admission was complicated by a marked reduction in her GCS (5/15) along with new-onset left-sided facial weakness and ptosis. On admission day 6, a diffusion‐weighted magnetic resonance imaging sequence (DWI‐MRI) revealed acute ischemic changes in the midbrain and bilateral thalami, which were consistent with the occlusion of AOP. The patient deteriorated very quickly and passed away during the same admission. This case illustrates the elusive clinical presentation of AOP infarction and underscores the urgent need for adequate neuroimaging to avoid any unwarranted medical treatment.

## Introduction

Bilateral thalamic infarcts constitute a rare and potentially underrecognized neuroradiological finding, likely secondary to their subtle and nonspecific clinical presentation [1]. Bilateral thalamic ischemia is mostly the result of an occlusion encompassing the artery of Percheron (AOP). The latter has been identified as an anatomical variant wherein a solitary thalamoperforating artery, typically arising from the posterior cerebral artery, supplies both paramedian thalami and occasionally portions of the rostral midbrain [2,3].

AOP infarcts represent approximately 0.1% of ischemic strokes and <5% of thalamic strokes [4]. A multitude of risk factors have been identified in relation to AOP infarction, including hypertension, diabetes, and atrial fibrillation [3,5]. A mean age of approximately 59 years and male predominance (57.7%) have been noted [4]. In contrast to the region-specific findings of a middle or posterior cerebral artery stroke, occlusion of the AOP has been associated with an atypical spectrum of clinical symptoms, including altered consciousness, speech and memory impairment, and oculomotor dysfunction [6,7].

Given the diagnostic challenges and the potential for misdirected management, an early utilization of relevant imaging techniques is critical in such cases. Radiologists have often debated the limitations in the use of magnetic resonance angiography (MRA) and digital subtraction angiography (DSA) in the detection of AOP infarcts due to the small arterial caliber and overlap with other vascular structures within the midbrain and thalamic areas [8]. Contrariwise, diffusion-weighted imaging magnetic resonance imaging (DWI-MRI) is critical in directly detecting thalamic infarcts given its high sensitivity to cytotoxic edema resulting from acute infarction [9]. The timing of MRI, however, remains critical and has the potential to alter the overall course of treatment and prognosis.

## Case presentation

**Figure 1:**
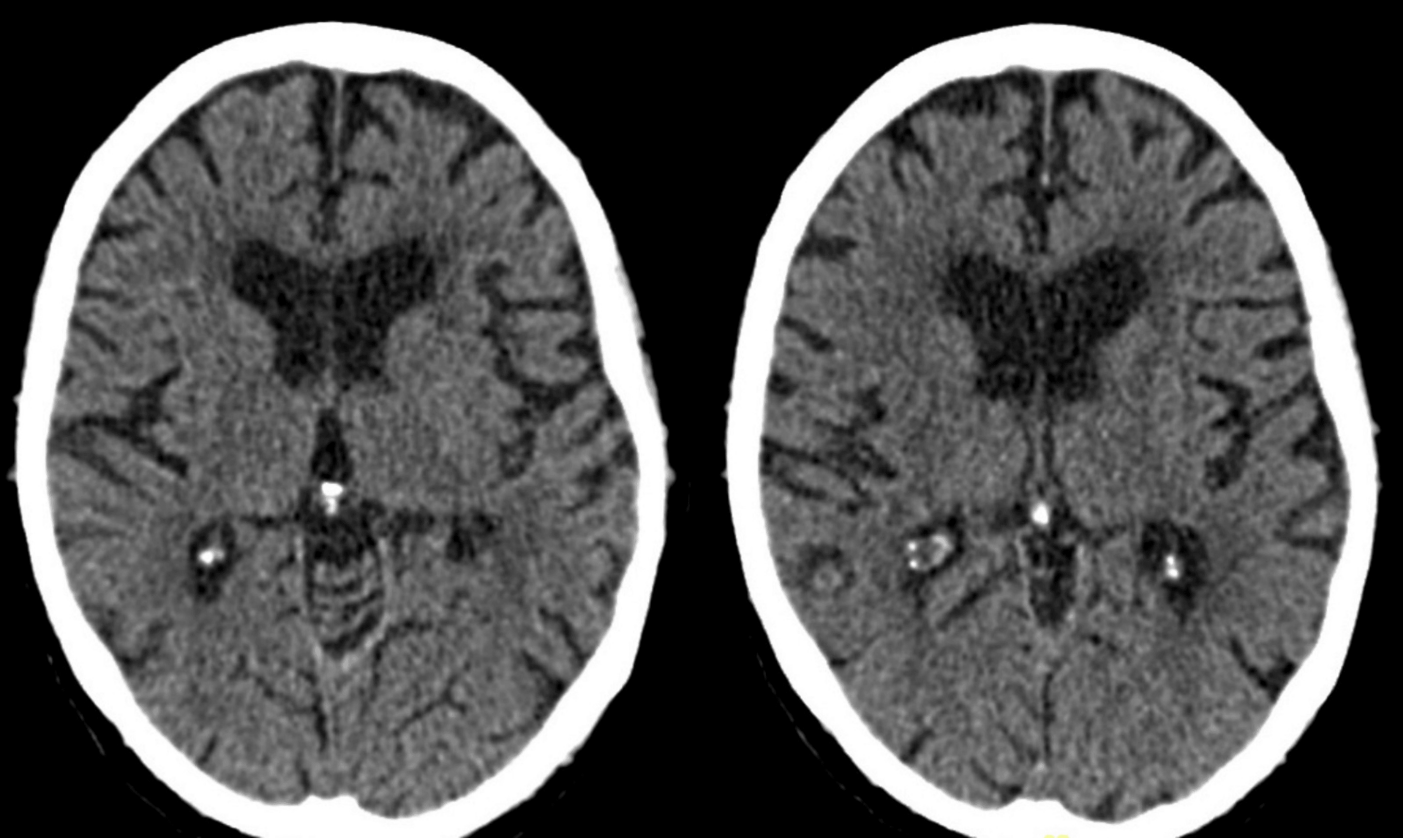
Non-contrast computed tomography (NCCT) head that shows no evidence of acute infarction in bilateral thalami.

An 86-year-old female presented after being found unresponsive at her residence in the morning. Her past medical history included type 2 diabetes mellitus, chronic kidney disease (CKD) stage IV, previously treated breast carcinoma, rheumatoid arthritis, and generalized cognitive decline. On examination, the Glasgow coma scale (GCS) score was 10/15 (E3V3M4), pupils were 4mm in diameter and bilaterally reactive. Blood sugar levels were normal. Chest examination revealed mild coarse bi-basal crepitations, while no significant findings were noted on cardiovascular and abdominal evaluation. Patient’s blood tests were unremarkable except mild anemia (Hb: 92 g/L) and an elevated C-reactive protein (CRP) level: 120 mg/L. Renal function was at the patient’s baseline of CKD stage IV (Table 1). Blood cultures later grew Gram-positive cocci. On day 1 of admission, a non-contrast computed tomography (NCCT) scan of the head ruled out intracranial hemorrhage (Figure 1).

Initially, an infection was the main diagnosis established (meningoencephalitis or sepsis) given the altered mental status and isolation of microbiological agents in blood cultures (later found to be non-significant). The patient was subsequently commenced on intravenous antibiotics (ceftriaxone and amoxicillin as per institutional protocol). Cerebrospinal fluid (CSF) analysis demonstrated a clear fluid with normal protein and glucose levels, low cell count (WBC: 6 per µL, RBC: 18 per µL). The Xanthochromia screen was also negative. CSF bacterial cultures viral multiplex polymerase chain reaction was negative (Table 1).

**Table 1:** Blood and cerebrospinal fluid (CSF) sample results for the case report. eGFR: Estimated glomerular filtration rate; CRP: C-reactive protein; PT: Prothrombin time; INR: International Normalized Ratio; CSF: Cerebrospinal fluid

Laboratory Parameters	Values	Normal Range
White cell count (10^9^/L)	7.8	4.0 - 11.0
Hemoglobin (g/L)	92	115 - 165
Platelets (10^9^/L)	302	150 - 400
Plasma glucose (mmol/L)	5.3	3.9-5.4
Sodium (mmol/L)	138	133 - 146
Potassium (mmol/L)	5.3	3.5 - 5.3
Chloride (mmol/L)	110	95 - 108
Urea (mmol/L)	12.1	2.5 - 7.8
Creatinine (umol/L)	185	45 - 84
eGFR result (mL/min/1.73 m^2^)	21	>60
Total bilirubin (umol/L)	6	0 - 21
Total protein (g/L)	68	60 - 80
CRP (mg/L)	120	<5
PT (seconds)	12.5	9.4 - 16.4
INR	1.06	0.8 - 1.25
CSF protein (g/L)	0.48	0.15 - 0.45
CSF glucose (mmol/L)	4.5	2.2 - 4.0
Net bilirubin absorbance (AU)	0.002	-
Net oxyhemoglobin absorbance (AU)	0.029	-

**Figure 2:**
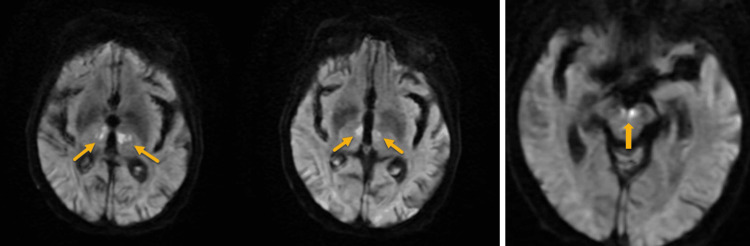
Diffusion-weighted sequence of MRI brain showing symmetrical diffusion restriction in the paramedian aspect of bilateral thalami and midbrain region, suggesting an acute infarction (as indicated by arrows).

During admission, the patient had further clinical deterioration and a marked drop in GCS to 5/15, accompanied by a new-onset left-sided facial weakness and ptosis. On day 6 of admission, a diffusion-weighted MRI scan was subsequently performed, which revealed diffusion restriction within bilateral thalami and midbrain (Figure 2). MRI findings shifted the diagnostic focus from infection to acute ischemic stroke, most consistent with occlusion of the AOP. Supportive management was continued, and the clinical course was closely monitored. Unfortunately, the patient’s condition deteriorated quickly, and she passed away on day 11 of admission.

## Discussion

Bilateral thalamic infarction secondary to occlusion of the AOP represents a diagnostic conundrum. The initial nonspecific presentation - characterized by reduced consciousness and subtle neurological findings - can easily be mistaken for other etiologies such as infectious or metabolic encephalopathies. In our patient, the early clinical picture and laboratory findings (elevated CRP and positive blood cultures) further clouded the diagnosis, leading to an initial management strategy targeting meningoencephalitis. The subsequent deterioration, highlighted by new focal deficits (e.g., left facial weakness and ptosis), raised clinical suspicion for a vascular event. DWI-MRI played a pivotal role by clearly delineating the ischemic changes in the thalamic and midbrain regions.

Bilateral thalamic lesions have many non-vascular mimics, including toxic-metabolic, inflammatory, or demyelinating encephalopathies, and this may blur the exact diagnosis unless there exists a low threshold of clinical suspicion [10]. A review by Atallah et al. (2024) reported heterogeneous clinical presentations encompassing symptoms such as an acute change in consciousness, hypersomnolence, or even coma, which may occur among up to three-quarters of patients with bilateral thalamic infarcts [4]. Hemiparesis and facial weakness have also been rarely documented, where the latter was also noted as part of our case findings [11]. Furthermore, oculomotor pathologies have been reported in up to 44% of cases and may be manifested as vertical gaze palsy, pupillary abnormalities, or ptosis [4]. To summarize, a triad of acute altered consciousness, ocular motor deficits, and memory impairment should raise suspicion for AOP infarction, all of which were reported in our case summary as well [3].

A prompt neuroradiological diagnosis is challenging since a routine CT brain is often unremarkable. In their case report, Kheiralla et al. (2021) found the first CT and even the bedside transcranial Doppler scans to be normal [3]. Contrariwise, Bhattarai et al. (2023) reported detection of bilateral thalamic hypodensities on the initial CT scan performed within 30 minutes of arrival [10]. Nonetheless, MRI remains the preferred imaging modality for confirming the diagnosis of thalamic infarction, where DWI and fluid-attenuated inversion recovery (FLAIR) MRI sequences typically reveal symmetric high-signal infarcts in the paramedian thalami. A classic “V-shaped” hyperintensity on axial FLAIR imaging can also be noted, often dubbed the V sign [12,13]. One Chinese series shows that this sign was discovered in approximately 17% of patients [8]. Given the small caliber of AOP, vascular imaging, including MR or CT angiograms, may fail to show an obvious AOP occlusion. Hence, it is noteworthy that the absence of an identifiable arterial blockage on angiography does not exclude AOP infarct [14]. In all cases, recognition of the characteristic bilateral thalamic pattern on MRI should raise a strong suspicion for AOP infarction.

The overall prognosis varies from case to case. Clinical outcomes in AOP infarction are variable and highly dependent on midbrain involvement. In one retrospective study by Zhang et al. (2022), approximately two-thirds of n = 23 patients had a good functional outcome (modified Rankin scale ≤2) at 3 months, while only ~26% completely returned to the functional baseline (Table 2). Notably, all patients who had rostral midbrain infarction remained significantly disabled [8]. In the case reported by Bhattarai et al. (2023), rostral midbrain infarction was also seen on the CT along with thalamic strokes, and the patient had a poor functional recovery [10]. Mesencephalic involvement was also noted on the MRI scan in our study and was associated with a significant functional decline. In contrast, isolated thalamic infarcts without midbrain ischemia may allow substantial recovery of neurological function, as noted in the study by Kheiralla et al. (2021), where the patient improved to near-baseline two weeks after stroke [3]. Nonetheless, brain MRI should be arranged promptly if an AOP infarct is suspected, since early thrombolysis (though only infrequently used in practice) may potentially improve the substantial morbidity associated with this rare stroke [8].

Table 2 cites findings from the most recent case reports and series (in the last 5 years) detailing the variability in overall neurological deficits and neuroimaging findings.

**Table 2:** Neurological and neuroimaging findings from case reports documenting AOP infarction. CT: Computed tomography; MRI: Magnetic resonance imaging; GCS: Glasgow coma scale; AOP: Artery of Percheron; DWI: Diffusion-weighted imaging; mRS: modified Rankin scale.

Authors	Study Type	Age/Gender	Presenting Complaint	Neurological Findings	CT Findings	MRI Findings	Clinical Outcomes
Lizwan and Sonu, 2023 [15]	Case report	90s/M	4-day history of increasing drowsiness, poor oral intake	Coma (GCS 7); no eye tracking; diffuse hyporeflexia	Acute infarcts in bilateral thalami, right occipital lobe, right cerebral peduncle	Bilateral paramedian thalamic infarcts (AOP territory)	Not reported (no outcome described)
Shams et al., 2021 [16]	Case report	58/M	Dysarthria and right facial weakness	Dysarthria; right facial palsy	Nil acute	Bilateral anterior thalamic infarcts	Symptoms fully resolved to baseline within 48 hours
Yamaguchi and Yakushiji, 2022 [17]	Case report	81/M	2-day history of impaired consciousness	Initially comatose (GCS 8); later somnolent, disoriented (GCS fluctuating 8–13)	Hypodensities in bilateral thalami, cerebellar hemispheres, right putamen, frontal lobes	Bilateral paramedian thalamic infarcts	Thrombolysis given; gradual improvement but persistent disorientation; transferred to sanatorium
Alaithan et al., 2023 [18]	Case report	58/F	Sudden confusion, speech difficulty, and right-sided weakness	Confusion; dysarthria; right hemiparesis	Left internal capsule hypodensity	Bilateral paramedian thalamic infarcts (AOP territory)	Thrombolysis given; improved with mild residual hemiparesis; discharged to rehabilitation
Lahnine et al., 2024 [19]	Case report	64/Unknown	Sudden coma onset (~24 h), GCS 11	Coma (GCS 11); no focal deficits noted initially	Bilateral thalamic hypodensities with ring enhancement	Bilateral thalamic and right midbrain infarcts with diffusion restriction and ring enhancement	Gradual improvement with patient alert and obeying commands by 48 hours; discharged to subacute rehab on dual antiplatelet therapy
Zhang et al., 2022 [8]	Retrospective study (n=23)	23–77 years; M=15, F=8	Different presentations included somnolence, coma, confusion, behavioral changes, and memory loss.	Reduced GCS, vertical gaze palsy, aphasia, dysarthria, and memory deficits	Nil acute in most patients; occasional thalamic hypodensity	Bilateral paramedian thalamic infarcts; some with rostral midbrain involvement; “V-sign” on DWI-MRI in select cases	Most patients recovered with mRS ≤2; some had long-term cognitive/behavioral issues
Macedo et al., 2022 [20]	Case series (n=8)	60–80 years; M =4, F=4	Different presentations included coma, hypersomnia, and cognitive dysfunction	Reduced GCS, ophthalmoplegia, hemiparesis, dysarthria, hypersomnia, and memory deficits	Bilateral thalamic hypodensities in a few cases, but mostly unremarkable	Symmetrical bilateral paramedian thalamic infarcts and frequent midbrain involvement	2/8 patients died; others recovered partially with some functional dependence or residual deficits

## Conclusions

Our case study underscores the importance of recognizing the classic triad of altered consciousness, oculomotor dysfunction, and memory impairment for patients suspected of bilateral thalamic infarction. A multidisciplinary approach incorporating meticulous clinical assessment and timely MRI can significantly improve diagnostic clarity in these challenging cases. Midbrain involvement, as evident in our patient, has been associated with poorer prognosis and limited neurological recovery. In such cases, early diagnosis may still play a pivotal role by guiding decisions regarding supportive care and informing prognostication.
